# Ordinary differential equations and Boolean networks in application to modelling of 6-mercaptopurine metabolism

**DOI:** 10.1098/rsos.160872

**Published:** 2017-04-12

**Authors:** Anastasia I. Lavrova, Eugene B. Postnikov, Andrey Yu. Zyubin, Svetlana V. Babak

**Affiliations:** 1 Immanuel Kant Baltic Federal University, A. Nevskogo st. 14A, Kaliningrad, Russia; 2 St Petersburg Research Institute of Phthisiopulmonology, Polytechnicheskaya st. 32, Saint-Petersburg, Russia; 3 Department of Theoretical Physics, Kursk State University, Radishcheva st. 33, Kursk, Russia

**Keywords:** kinetic modelling, drug metabolism, Boolean networks

## Abstract

We consider two approaches to modelling the cell metabolism of 6-mercaptopurine, one of the important chemotherapy drugs used for treating acute lymphocytic leukaemia: kinetic ordinary differential equations, and Boolean networks supplied with one controlling node, which takes continual values. We analyse their interplay with respect to taking into account ATP concentration as a key parameter of switching between different pathways. It is shown that the Boolean networks, which allow avoiding the complexity of general kinetic modelling, preserve the possibility of reproducing the principal switching mechanism.

## 1. Introduction

6-Mercaptopurine (6-MP) is one of the important chemotherapy drugs used for treating acute lymphocytic leukaemia (ALL). It belongs to the class of medications called purine antagonists and works by stopping the growth of cancer cells. 6-MP undergoes extensive metabolic intracellular transformations that result in the production of thionucleotides and active metabolites, which have cytotoxic and immunosuppressive properties leading to various acute side-effects such as kidney effects, hepatotoxicity, pancreatitis and neuropathy.

**Figure 1.**
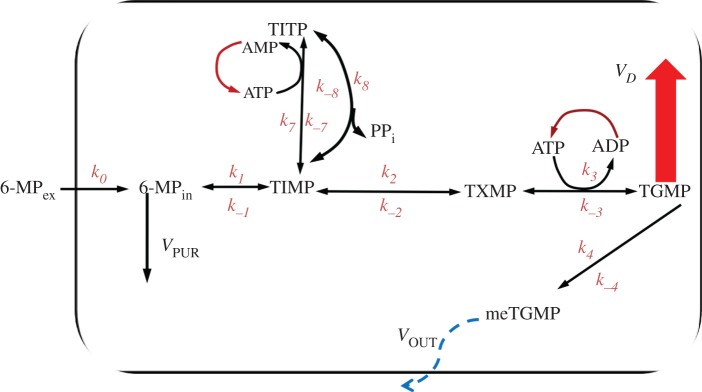
Simplified scheme of 6-MP metabolism. *k*_*i*_/*k*_−*i*_, kinetic constants of forward-back reactions; 6-MP_ex_, 6-MP_in_, mercaptopurine outside and inside of cell, TIMP; TITP, 6-thioinosine-5’-monophosphate and triphosphate; TXMP- 6-thioxanthine 5’-monophosphate; TGMP, 6-thioguanosine monophosphate; meTGMP, 6-methylthioguanosine monophosphate; ATP, ADP and AMP, adenosine tri-, di- and monophosphates, respectively; *V*_*D*_, *V*_PUR_ and *V*_OUT_, common fluxes describing incorporation into DNA and RNA of cells, inhibition of purine biosynthesis *de novo* and outflux to environment, respectively.

The conversion of 6-MP according to the metabolic scheme presented in figure 1 involves several small pathways [1]. The desired pathway results in the formation of 6-thioguanosine monophosphate (TGMP) that could be incorporated (via some metabolic transformations) into DNA and RNA, leading to tumour cell death [2–4] and successful treatment of ALL. The catabolic pathways regulated by the enzyme mercaptopurine methyltransferase (TPMT) lead to the production of various methyl-mercaptopurines affecting purine biosynthesis [2], which leads to treatment failure in most cases [3]. The transformation (figure 1) of 6-thioinosine-5’-monophosphate (TIMP) to 6-thioinosine-5’-triphosphate (TITP) is an additional pathway, which results in the accumulation of cytotoxic products (TITP and TDTP) and slows the production of TGMP. Since the realization of each pathway depends on the properties of the enzymes that are considered the main regulators of the ratio of activated to inactivated metabolites, then polymorphisms in the corresponding genes can lead to drug tolerance during ALL therapy [2,5]. On the other hand, energetic balance disturbance connected with mitochondrial dysfunction can play a crucial role in the appearance of side-effects and treatment failure [6,7]. So, it was shown that the level of ATP drastically decreases owing to cell necrosis that could be caused by hypoglycaemia accompanied by loss of cells’ ability to take up glucose [8–11]. Also, ATP is consumed during the transfer of drugs through special membrane transporters [12].

Besides experimental studies, enzyme activities in 6-MP metabolism and regulation effects have been exposed to numerical simulations and mathematical modelling [2,3,13]. These detailed semi-mechanistic models involve various compartments of the human organism, from cells to organs, to describe side-effects depending on the drug dose, which allows for forecasting of the optimal dose for successful treatment. However, these models were focused on the properties of regulating enzymes and exclude energy metabolism, which may play a crucial role in the occurrence of side-effects [7]. Moreover, the large-scale networks of interacting components require the adjustment of a large number of kinetic constants, which prevents understanding of principal mechanisms and key parameters of switching between the pathways in 6-MP metabolism.

This is why a different approach proposed by Glass & Kaufmann [14] has gained popularity: Boolean networks (for reviews of the state of the art, see e.g. [15–17]). A Boolean network represents a graph whose nodes can take values 0 (inactive) or 1 (active) and edges are matched to the rules of Boolean logic. Their evaluation with respect to the previous logical states of the nodes determines the consequent state of the network’s nodes.

Certainly, if ordinary differential equation (ODE)-based models are over-complicated, the Boolean networks are often over-simplified. For example, sometimes their over-simplicity requires the introductions of tricks, somewhat artificial, such as over- and under-self-expressed nodes with the values 1±0.5 [18]. This situation calls for some hybrid models, which should have the best aspects of both approaches [17,19].

This problem is closely connected with the question about the interplay of the ODEs modelling the scheme of kinetic reactions and the Boolean networks simulating the activity of the reactants. This challenge has motivated a number of works; one can mention the pioneering article [20], as well as recent developments [21,22]. However, the approaches considered in these works deal with the processes that exhibit sharp transitions. In other words, such ODEs correspond to the high-order Hill kinetics and the extraction of fast processes is possible. It is a natural situation for the gene/protein networks, but the component kinetics of biochemical metabolic networks is smoother.

Thus, one of the goals of the present work is an attempt to overcome this difficulty using a certain freedom provided by probabilistic Boolean networks [23]: a set of Boolean networks, each of them corresponding to a different pathway, and a choice between them is determined by potential interactions between underlying biological components and their uncertainties. In our case, we intend to introduce a dynamic continual variable, which will control such switching.

## 2. Material and methods

Numerical simulations have been carried out using the software package MATLAB 2009b granted by the Baltic Federal University and the Kursk State University.

## 3. Kinetic ordinary differential equation model

To describe the principal dynamics of the 6-MP metabolic transformations and to single out the key nodes of this ‘metabolic chain’, we have proposed a model which describes the simplified kinetic scheme shown in figure 1. The dimensional model does not detail the dynamics of each enzyme but involves ATP concentration as a key player of energy metabolism. We suggest that the influx of ATP is constant due to the energetic metabolism of the cell, which provides a constant pool of ATP production [24–26].

Concerning the initial conditions of the model, two values were defined, the concentrations of 6-MP_ex_ and ATP obtained experimentally [27] and, according to the protocol BFM-ALL 2000 [28], developed for acute leukaemia treatment. The concentrations of other metabolites have been taken to be zero at the starting point. Concerning the kinetic constants, they were chosen with respect to the described (database brenda-enzymes.org) enzyme dynamics of all metabolite transformations and to the experimental data presented previously [27,29–31].

As a result, the system of ODE, corresponding to the simplified kinetic model, can be written as follows:

$$\begin{array}{rl} \frac{d}{dt}\;{\mathrm{MP}}_{\mathrm{ex}} & =-k_{0}\;{\mathrm{MP}}_{\mathrm{ex}},\\ \frac{d}{dt}\;{\mathrm{MP}}_{\mathrm{in}} & =-(V_{\mathrm{PUR}}+k_{1})\;{\mathrm{MP}}_{\mathrm{in}}+k_{0}\;{\mathrm{MP}}_{\mathrm{ex}}+k_{-1}\mathrm{TIMP},\\ \frac{d}{dt}\;\mathrm{TIMP} & =k_{1}\;{\mathrm{MP}}_{\mathrm{in}}+k_{-8}\;\mathrm{TITP}-(k_{2}+k_{7}\;\mathrm{ATP}+k_{-1}+k_{8}\mathrm{PP})\mathrm{TIMP}\\ & \;+k_{-2}\;\mathrm{TXMP}+k_{-7}\;\mathrm{TITP}\cdot \mathrm{AMP},\\ \frac{d}{dt}\;\mathrm{TXMP} & =k_{2}\;\mathrm{TIMP}-k_{3}\;\mathrm{TXMP}\cdot \;\mathrm{ATP}-k_{-2}\;\mathrm{TXMP}+k_{-3}\;\mathrm{TGMP}\cdot \mathrm{AMP}\cdot \;\mathrm{PP},\\ \frac{d}{dt}\;\mathrm{TGMP} & =k_{3}\;\mathrm{TXMP}\cdot \;\mathrm{ATP}-(k_{4}+V_{D})\;\mathrm{TGMP}-k_{-3}\;\mathrm{TGMP}\cdot \mathrm{AMP}\cdot \mathrm{PP}+k_{-4}\mathrm{meTGMP},\\ \frac{d}{dt}\;\mathrm{meTGMP} & =k_{4}\;\mathrm{TGMP}-V_{\mathrm{OUT}}\;\mathrm{meTGMP}-k_{-4}\;\mathrm{meTGMP},\\ \frac{d}{dt}\;\mathrm{TITP} & =k_{8}\;\mathrm{TIMP}\cdot \mathrm{PP}-k_{-8}\;\mathrm{TITP}+k_{7}\;\mathrm{TIMP}\cdot \mathrm{ATP}-k_{-7}\;\mathrm{TITP}\cdot \mathrm{AMP},\\ \frac{d}{dt}\;\mathrm{ATP} & =-k_{7}\;\mathrm{TIMP}\cdot \mathrm{ATP}+k_{-3}\;\mathrm{TGMP}\cdot \mathrm{AMP}\cdot \mathrm{PP}-k_{3}\;\mathrm{TXMP}\cdot \mathrm{ATP}+k_{-7}\;\mathrm{TITP}\cdot \mathrm{AMP},\\ \frac{d}{dt}\;\mathrm{AMP} & =-k_{-3}\;\mathrm{TGMP}\cdot \mathrm{AMP}\cdot \mathrm{PP}+k_{3}\mathrm{TXMP}\cdot \mathrm{ATP}+k_{7}\mathrm{TIMP}\cdot \mathrm{ATP}-k_{-7}\;\mathrm{TITP}\cdot \mathrm{AMP}\\ \;\mathrm{and}\;\;\frac{d}{dt}\;\mathrm{PP} & =-k_{8}\;\mathrm{TIMP}\cdot \mathrm{PP}+k_{-8}\mathrm{TITP}-k_{-3}\;\mathrm{TGMP}\cdot \mathrm{AMP}\cdot \mathrm{PP}+k_{3}\;\mathrm{TXMP}\cdot \mathrm{ATP}. \end{array}$$

In our simulations, the kinetic constants corresponding to the biophysically relevant dynamics were determined as *k*_0_=5*d*^−1^, *k*_1_=10*d*^−1^, *k*_2_=10*d*^−1^, *k*_3_=5*M*^−1^*d*^−1^, *k*_4_=0.00001*d*^−1^, *k*_7_=0.01*d*^−1^, *k*_8_=0.5*M*^−1^*d*^−1^, *k*_−7_=1*M*^−1^*d*^−1^, *k*_−1_=0.01*d*^−1^, *k*_−2_=4*d*^−1^, *k*_−3_=0.01*M*^−2^*d*^−1^, *k*_−4_=0.1*d*^−1^, *k*_−8_=0.01*d*^−1^, *V*_PUR_=0.01*d*^−1^, *V*_*D*_=0.9*d*^−1^, *V*_OUT_=0.0001*d*^−1^, where *M* means μmol ml^−1^, and *d* means days.

The initial concentrations were equal to zero for all variables except for the fixed value of MP_ex_(0)=0.68 μmol ml^−1^ and ATP(0), whose value plays the role of a control parameter.

## 4. A Boolean network mimicking the key dynamical processes

### 4.1. Network construction

The simplified metabolic network described above allows for the representation in terms similar to the probabilistic Boolean network. The resulting network consists of five nodes {*y*_*i*_}, *i*=1 … 5 and the threshold-based rule *A*(*α*_*j*_) for the choice between possible pathways. The value of the continuous control parameter *α*_*j*_ could be non-stationary depending on the iteration number *j*. These rules for *α*_*j*_ and *A*(*α*_*j*_) replace ODEs, which govern the dynamics of reactions connecting ATP, ADP and AMP, i.e. allow for reducing the three corresponding differential equations to one.

The correspondence of these nodes to the metabolites and the transition rules for the parallel update of the states are presented in table 1. Note also that the irreversible degradation of MP_ex_ is modelled as an initial condition for the node *y*_1_ corresponding to MP_in_, which is directly influenced by MP_ex_ in the full-scale ODE-based kinetic model. In addition, we excluded the dynamics of the metabolite meTGMP, which is a by-product with respect to the main metabolic path of interest.

**Table 1.** The nodes and transition rules for the considered Boolean network.

node	metabolite	rules of interactions and updating
*y*_1_	6-MP_in_	starting node activated, when 6-mercaptopurine enters the cell. It activates TIMP and then will be deactivated
*y*_2_	TIMP	this node is activated by 6-MP_in_ *or* by TITP and can activate nodes TXMP or TITP depending on a chosen pathway (the choice is governed by the variable *α*); it is deactivated after this
*y*_3_	TXMP	this node is activated by TIMP and can activate TGMP or TIMP depending on a chosen pathway (the choice is governed by the variable *α*); it is deactivated after this
*y*_4_	TGMP	this node indicate the target output, is activated by TXMP and deactivated after the completed output
*y*_5_	TITP	this node is activated by TIMP within one of the possible pathways and activates TIMP; it is deactivated after this
*α*	ATP	the continual parameter, which governs a choice of pathways as follows: *if α*<0.5, then then the irreversible activation TXMP→TGMP is chosen; the reversible transition TXMP⇌TGMP holds otherwise; *if α*<0.75 the pathway through TXMP is chosen, the pathway through TITP holds otherwise. The parameter *α* is non-stationary and satisfies the decay kinetics $\dot{\alpha }=-\kappa \alpha$ if the process goes through the TIMP pathway

The realization of this via Boolean and conditional operators reads as follows (here the states of the nodes are grouped into the matrix *y*(*j*,*i*)):


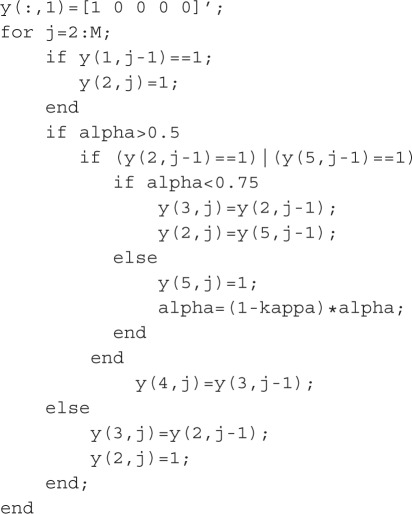


Here ‘=’, ‘==’, and ‘|’ operators denote the assignment, the equality, and OR respectively. The degradation conditions mentioned in table 1 fulfil automatically during the transition to a subsequent iteration step if the target node was not activated explicitly. It is a specific feature of the programming language, which initializes new arrays as filled by zeros. Note that the code represented above can be evaluated straightforwardly using MATLAB or other software that supports MATLAB-like syntax (e.g. OCTAVE, FreeMat) if supplied with initial conditions and a value of the decay parameter. The last one is introduced via the simplest discretization of the equation $\dot{\alpha }=-\kappa \alpha$ via the Euler scheme with the unit time step (i.e. in accordance with the assumed step of network node updating): *α*_*j*+1_=(1−*κ*)*α*_*j*_. This degradation rule is based on the kinetics of the processes ATP→ADP+P and ATP→AMP+2P with constant coefficients that take into account the simplification reasonable for Boolean network modelling, because Boolean networks operate with two-value constant indicators of node activity (0 or 1) instead of concentrations, in contrast to the detailed ODE kinetics where the terms such as *k*_7_TIMP vary.

The initial conditions may be stated as


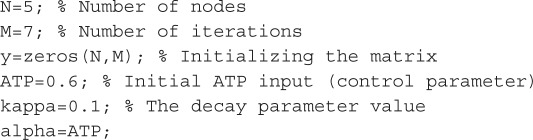


It should be pointed out that the scheme acts deterministically and its evolution of states is completely determined by the rules of the node states updating and the initial conditions. Thus our approach differs from the probabilistic Boolean networks [23] although uses their key property of introducing a continual parameter, which controls the choice between available pathways.

### 4.2. Simulation results

**Figure 2.**
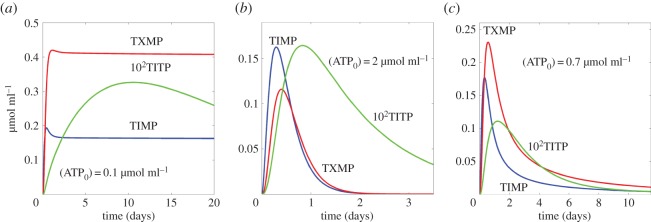
(*a*–*c*) Dependence of the dynamics of the metabolic chain on the initial concentration of ATP. Red, blue and green curves show the time course of concentrations of the basic metabolites as denoted. All simulations are evaluated in the same time scale but (*b*) and (*c*) demonstrate shorter axis intervals with the goal to make the peaks more distinguishable. Note that the concentration of TITP is multiplied by 100 for better visibility in the given scale.

First of all, we detect that the increase of metabolite concentrations occurs sequentially for one node after another along the ‘metabolic chain’. As an example, see figure 2, where the peak of TIMP concentration always occurs before the peak of TXMP concentration as these nodes are located on the direct path (horizontal line) from MP_ex_ to TGMP (figure 1). Thus, this confirms the possibility of using a Boolean network with the prevalence of sequential irreversible switches of states although the detailed ODE-based kinetic system also includes reversible individual steps.

It is revealed that TIMP is the key node of the reaction cascade since it provides two pathways, slow and fast, that also defines the blockage of the slow path interacting with ATP. Here we can define the concentration of ATP as the ‘key player’ in 6-MP metabolism, which regulates the transitions into two main points: the metabolic pathway of TITP production (the chain’s branch from TIMP) and the transition TXMP→TGMP. Such behaviour is traceable in figure 2, which shows the dynamics of both metabolites, TXMP and TITP, following TIMP on the mentioned two branches.

The simulations of this kinetic model show that small concentrations of ATP lead to the blockage of the metabolic chain in the node TIMP (figure 2*a*): further conversion stops since the concentration of TXMP is constant up to the end of simulations. Large concentrations of ATP result in the competition between the production of TITP (the end product of the branch) and TGMP (the product of the chain; figure 2*b*,*c*). Note that here we extracted shorter parts of the full simulated curve for better visibility of all details.

Very large concentrations of ATP (figure 2*b*) result in a higher level of TITP concentration, which decays sufficiently slower than the spikes of TIMP and TXMP. In addition, the concentration of TXMP is smaller in comparison with TIMP.

The reverse situation occurs for intermediate values of ATP concentration. A set of simulations shows that visually the concentration of ATP that shifts the pathway to higher production of TGMP is equal to about 0.7 μmol ml^−1^, as presented in figure 2*c*. Here, the peak of high TXMP concentration corresponds to the decay in TIMP dynamics. Moreover, TIMP dynamics looks like a pulse that means fast up and down to low concentrations, while the TXMP dynamics is much slower, which leads to the switch of pathway and provides a cumulative effort for producing the useful target product (TGMP). The concentration of TITP also drastically decreases in comparison with the case when ATP=2 μmol ml^−1^. In addition, TITP concentration decays within the same time range as TXMP, not slower.

The same situation can be observed in the simulation of Boolean networks. Table 2 represents the results of simulations evaluated for a set of increasing initial values (ATP) of the control parameter *α*. Although the Boolean networks approach does not operate with the concentrations by its definition, we chose the initial values of this parameter mimicking the numerical range considered within the ODE model. The results capture all principal features of the dynamics for the simulated network. Note that the first two steps are the same for all cases since the activation of TIMP by 6-MP_in_ is unconditional. The different pathways are realized during the next iterations only.

**Table 2.** The evolution of network states for various different values of the control parameter.

ATP=0.2							
*j*	1	2	3	4	5	6	7
*y*_1_	1	0	0	0	0	0	0
*y*_2_	0	1	1	1	1	1	1
*y*_3_	0	0	1	1	1	1	1
*y*_4_	0	0	0	0	0	0	0
*y*_5_	0	0	0	0	0	0	0

ATP=0.6							
*j*	1	2	3	4	5	6	7
*y*_1_	1	0	0	0	0	0	0
*y*_2_	0	1	0	0	0	0	0
*y*_3_	0	0	1	0	0	0	0
*y*_4_	0	0	0	1	0	0	0
*y*_5_	0	0	0	0	0	0	0

ATP=0.8							
*j*	1	2	3	4	5	6	7
*y*_1_	1	0	0	0	0	0	0
*y*_2_	0	1	0	1	0	0	0
*y*_3_	0	0	0	0	1	0	0
*y*_4_	0	0	0	0	0	1	0
*y*_5_	0	0	1	0	0	0	0

ATP=0.9							
*j*	1	2	3	4	5	6	7
*y*_1_	1	0	0	0	0	0	0
*y*_2_	0	1	0	0	1	0	0
*y*_3_	0	0	0	0	0	1	0
*y*_4_	0	0	0	0	0	0	1
*y*_5_	0	0	1	1	0	0	0

For ATP=0.2, the dynamics is blocked at the transition from TXMP to TGMP. Instead of the forward activation, the process goes back reactivating the node corresponding to TIMP. At the same time, since this reaction is reversible, the reactivation of TXMP occurs, etc. Thus, the system reaches a steady state, which is reflected in unit values of the nodes *y*_2_ and *y*_3_ spreading ad infinitum.

The value ATP=0.6 corresponds to the situation where the pathway TXMP→TGMP is allowed but the pathway leading to TITP is blocked. As a result, the transition process is direct and straightforward: the nodes *y*_1_–*y*_4_ are activated sequentially during the four sequential iterations. When *y*_4_ is activated, this means that the target substance is released, and all nodes switch off to zeros in the absence of a new influx into *y*_1_. Thus, the process under study is completed during four time steps, when the system reaches the attractor y(:,4)=[0 0 0 1 0]’; the other columns *j*=5 … 7 originate simply from the initial initialization of the matrix y as a 5×7 rectangular structure filled by zeros.

Both the values ATP=0.8 and ATP=0.9 exceed the threshold value of *α*=0.75. Whence, the pathway to TITP is available now. It is reflected as *y*_3_=0 but *y*_5_=1 at the third iteration, i.e. the pathway is changed. However, there is a difference in the further time evolution of the network’s states for these two cases. Namely, the table corresponding to ATP=0.8 demonstrates the activation of *y*_3_ (i.e. the backward transition TITP→TIMP) during the next iteration and the consequent sequential node activation along the pathway TIMP→TXMP→TGMP. On the other hand, these step are delayed in the case of ATP=0.8: both third and fourth iterations contain *y*_5_=1 and *y*_*i*_=0, *i*=1 … 4 only. Such a behaviour originates from the introduced non-stationarity of the control parameter *α*, which resembles the concentration of ATP. As was discussed above, the TIMP→ TITP pathway is an ATP-consuming process. Thus, each iteration corresponding to this pathway diminishes *α* while it will cross the threshold *α*=0.75 from above. Furthermore, this pathway will be blocked. The cases ATP=0.8 and ATP=0.9 require one and two iterations for this decay of *α*, respectively. Larger values of ATP will result in larger delays.

Finally, we should note that the discussed results are deterministic since they correspond to individual realizations. However, it allows several generalizations leading to probabilistic Boolean networks in a general case. For example, one can generate an ensemble of realizations with ATP randomly distributed with respect to some appropriate probability distribution. The output will be a distribution of the node values during the iterations. In this case, each individual trajectory of states will be deterministic but the choice between the paths will include certain randomness depending on the relationship between the initial value of ATP and the threshold values of *α*. Another way is to include an additive random noise into the equation governing the dynamics of *α*. But these procedures are outside of the direct goals of this work.

## 5. Discussion

It is known that the methylation of 6-MP resulting in the formation of intermediate metabolites occurs at a low concentration of intracellular ATP (0.1 μmol ml^−1^). Simultaneously, the concentrations of TIMP and TXMP remain at a prolonged constant level. At these conditions, the production of the final metabolite, TGMP, slows down. As a result, the therapeutic efficiency also diminishes but the risk of toxic action grows since intermediate metabolites of 6-MP inhibit the biosynthesis of *de novo* purines. Thus, a decreasing intracellular ATP pool in T-lymphocytes results in higher toxicity and lower efficiency of this drug [7,32].

Our results model an effect of high initial concentration of ATP on the metabolism of 6-MP. They show that ATP concentrations of 2 μmol ml^−1^ produce high concentrations of the intermediate metabolite TIMP, which indicates an incomplete metabolism of the drug accompanied by the production of TGMP insufficient for the therapeutic action. Therefore, we suggest that intensive TIMP formation plays a role as a marker indicating the accumulation of toxic final metabolites at a high level of intracellular ATP.

The concentration change of ATP is a key factor for the energy exchange deficit accompanied by mitochondrial dysfunction [33]. This results in decreased therapeutic effect of drugs during tumour treatment. It has been shown that glycolysis inhibition by an attenuation of glucose consumption in cells leads to diminishing of the ATP level and, finally, results in tumour cell death. However, the process of energy deficiency is reversible since the cell activates another pathway that supports ATP accumulation and the cell will recover its function.

The results obtained using our model argue that the optimal initial ATP concentration is equal to 0.7 μmol ml^−1^. It corresponds to the situation when 6-MP metabolism is a complete process resulting in both production of therapeutically active products and the reduction of the pool of toxic intermediate products.

During administration of cytotoxic drugs according to the protocol BFM ALL 2000 [28], it is expedient to keep the concentration of intracellular ATP within the middle range to prevent risk of adverse drug reactions instead of an artificial inhibition of energy metabolism [33].

The clinical indication of low ATP concentration is acidosis by lactate accumulation [33]. This leads to mitochondrial dysfunction and an additional toxic effect. Higher ATP concentrations inhibit glycolysis resulting in glucose accumulation, glucose tolerance, and, indirectly, in the development of cardiomyopathy [34].

Thus, we hypothesize that the maintenance of ATP at an intermediate level is the necessary condition to reach a complete therapeutic effect and diminish toxicity of the chemotherapy process.

## 6. Conclusion and outlook

In this work, we have analysed the dynamic behaviour of the metabolic pathways of 6-MP with a focus on revealing the key parameter that switches between the two principal ‘branches’, slow and fast. The results of simulations based on the system of ordinary equations indicate that ATP is the desired ‘key player’ in 6-MP metabolism. This conclusion is supported by a number of phenomenological observations presented in the modern biomedical literature and allows for quantitative clarification of the underlying processes.

Based on the results of ODE modelling, we have reformulated the problems in terms of the hybrid Boolean network, which can be considered as a deterministic analogue of the probabilistic Boolean networks. This approach is much simpler in realization since it does not require the knowledge of multiple kinetic parameters but, at the same time, adequately reproduces the key details of the switching principal dynamic regimes as a choice between different possible pathways. Therefore, it can be scaled to a more detailed picture of metabolite interactions in future research of the studied process.

We also need to highlight the crucial feature introduced into the construction of the network: a non-stationary continual parameter, which governs the switching process. Such an approach, which has demonstrated its effectiveness in the considered case study, opens new perspectives for ‘hybridizing’ the continual (ODE-based) and discrete (Boolean) approaches to metabolic modelling. In contrast to previous works [20–22], which considered Boolean networks only as a limiting case of continual-time kinetic processes (in fact, mimicking the switching between unstable stationary states by node activity), the introduction of non-stationarity into the probabilistic parameter allows the consideration of smoother transitions, and, in principle, even an activity of small sub-networks with a small number of kinetic constants considered as building blocks for a large Boolean network.

## Data accessibility

There is no supporting material or special data since all equations, parameters used and algorithm are presented in the main text of the work and could be reproduced by anyone. Thus, paper contains complete self-sufficient information and does not need any additional data files.
